# The Interplay between Cannabinoid Receptors and Microglia in the Pathophysiology of Alzheimer’s Disease

**DOI:** 10.3390/jcm12237201

**Published:** 2023-11-21

**Authors:** Rebecca Ferrisi, Francesca Gado, Caterina Ricardi, Beatrice Polini, Clementina Manera, Grazia Chiellini

**Affiliations:** 1 Department of Pharmacy, University of Pisa, 56126 Pisa, Italy; clementina.manera@unipi.it; 2 Department of Pharmaceutical Sciences, University of Milan, 20133 Milano, Italy; francesca.gado@unimi.it; 3 Department of Pathology, University of Pisa, 56126 Pisa, Italy; c.ricardi@student.unisi.it (C.R.); grazia.chiellini@unipi.it (G.C.)

**Keywords:** cannabinoid receptors, microglia, neuroinflammation

## Abstract

Alzheimer’s disease (AD) is characterized by massive neuronal death, brain atrophy, and loss of neurons and synapses, which all lead to a progressive cognitive decline. Neuroinflammation has been recently identified as one of the main causes of AD progression, and microglia cells are considered to have a central role in this process. Growing evidence suggests that cannabinoids may be used as preventive treatment for AD. An altered expression of the endocannabinoids (eCBs) and their receptors (CBRs) is reported in several neurodegenerative disorders, including AD. Moreover, the modulation of CBRs demonstrated neuroprotective effects in reducing aggregated protein deposition, suggesting the therapeutic potential of natural and synthetic CBR ligands in the treatment of neurodegenerative proteinopathies. Here, we review the current knowledge regarding the involvement of CBRs in the modulation of microglia activation phenotypes, highlighting the role of neuroinflammation in the pathogenesis of neurodegenerative diseases, like AD. We also provide an overview of recently developed candidate drugs targeting CBRs that may afford a new innovative strategy for the treatment and management of AD.

## 1. Introduction

### 1.1. A Glimpse into the Architecture of the Endocannabinoid System

The endocannabinoid system (ECS) is a complex and ubiquitous lipid signaling system implicated in a multitude of physiological and pathological processes; the ECS is involved in the development and regulation of the central and peripheral nervous system and the modulation of immune and endocrine systems [1]. The broad participation in these vital processes gives the ECS an enormous therapeutic potential that remains to be fully exploited.

The ECS employs a large network of components (receptors, ligands, and enzymatic machinery molecules) that cooperate for the maintenance of tissue and cellular homeostasis. More precisely, this system is composed of two specific cannabinoid receptors (CB1 and CB2 receptors), and their endogenous ligands, namely endocannabinoids (eCBs). The eCBs are a class of lipid-based neurotransmitters, including anandamide (AEA) and 2-arachidonoylglycerol (2-AG) [2,3]. Parts of the ECS are also the main enzymes responsible for eCBs biosynthesis, i.e., N-acyl-phosphatidylethanolamine-phospholipase (NAPE-PLD) and diacylglycerol lipase (DAGL), as well as degradation, including fatty acid amide hydrolase (FAAH) [4] and monoacylglycerol lipase (MAGL) [5].

Unlike most other neurotransmitters and hormones, eCBs are not stored in vesicles but rather are synthesized from membrane lipids in response to specific signals. They are produced “on-demand” in response to increased intracellular Ca^2+^ concentration and released into the synaptic cleft before acting retrogradely on pre-synaptic CBR to inhibit neurotransmitter release [6]. AEA has a strong affinity for the CB1 receptor (CB1R), acting as a partial agonist, whereas 2-AG displays only a moderate to low affinity for both receptor subtypes, but acts as a full agonist [7]. AEA is normally synthesized from NAPE, which is formed by the transfer of arachidonic acid from the *sn-1* position of a donor phospholipid to phosphatidylethanolamine by N-acyltransferase. Hydrolysis of NAPE by an N-acylphosphatidylethanolamine-hydrolyzing phospholipase D produces anandamide. There are, however, alternative pathways of anandamide synthesis. The principal enzyme for the degradation of anandamide is FAAH [8]. The best-studied synthetic pathways for 2-AG involve the activation of a phospholipase C, which hydrolyzes inositol phospholipids at the *sn-2* position to produce diacylglycerol. The hydrolysis of diacylglycerol via *sn-1*-selective DAGL-α (and potentially DAGL-β) then leads to the formation of 2-AG [9]. There are also other pathways for the synthesis of 2-AG. 2-AG is mostly degraded by MAGL, but in mouse brains, about 15% of the degradation is by the enzymes α/β hydrolase domain-containing protein-6 (ABHD-6) and ABHD-12. 2-AG can also be oxygenated by cyclooxygenase-2 (COX2) to form biologically active prostaglandin glyceryl esters, which regulate inflammation [10].

### 1.2. Focus on Cannabinoid Receptors CB1R and CB2R

The two primary CBR subtypes, namely CB1R and CB2R, are *class A* members of the GPCR superfamily and, as such, they are seven-transmembrane (TM) spanning metabotropic receptors.

Human CB1R and CB2R display approximately 44% homology in their amino acid sequence and 68% similarity in the TMs. While CB1R is encoded by the *CNR1* gene and consists of 472 amino acids, CB2R is instead encoded by the *CNR2* gene, which is composed of 360 amino acids, and it shows comparatively greater interspecies heterogeneity than CB1R [11,12].

The two CBR subtypes differ in tissue distribution, as well as in signaling mechanisms. Since CB1R was found in the central nervous system (CNS), this aspect led to the “false myth” that the function of this receptor was limited to the brain. On the other hand, CB2R was mistakenly referred to as a “peripheral receptor”. However, later evidence has “rewritten” their distribution, reaffirming their ubiquity in both central and peripheral districts.

In the CNS, a high concentration of CB1R is reported in the cerebral cortex, basal ganglia, hippocampus, and cerebellum, where it controls a variety of physiological processes including memory, cognition, motor function, and pain transmission. Some regions of the brain have a moderate density (hypothalamus and spinal cord), while others, such as the thalamus and brainstem, exhibit low levels of CB1R [13]. Within the neuron, CB1R is often localized in axon terminals, and its activation leads to the inhibition of transmitter release. The consequence is the inhibition of neurotransmission via a presynaptic mechanism. Inhibition of glutamatergic, GABAergic, glycinergic, cholinergic, noradrenergic, and serotonergic neurotransmission has been observed in many regions of the CNS. In the peripheral nervous system, CB1R-mediated inhibition of adrenergic, cholinergic, and sensory neuroeffector transmission has been frequently observed. Besides the well-known plasma membrane localization of CB1R, which is the typical distributional pattern of GPCRs, multiple studies reported predominant intracellular localization of CB1R in diverse types of cells, including cultured hippocampal neurons, undifferentiated neuronal cells, and transfected non-neuronal cells [14].

Notably, in addition to neurons, CB1R is also expressed in astrocytes, oligodendrocytes, and microglia [15]. Peripherally, CB1R is expressed in a wide variety of districts, including the spleen, lung, liver, heart, vasculature, adipose tissue, the gastrointestinal (GI) tract, the spinal cord, the adrenal and thyroid glands, and reproductive organs [13].

CB2R is largely distributed in immune system cells, such as macrophages, leucocytes, spleen, tonsils, and the thymus, where it regulates cytokine release and cell migration [16]. Later research showed that CB2R is present in many other systems, including the CNS, cardiovascular and respiratory systems, gastrointestinal tract, liver, skeletal muscle, bone, and the reproductive system [17]. Regarding its central distribution pattern, functional expression of CB2R has been recently detected in various districts of the brain, including the striatum [18], cortex, amygdala, cerebellum [19], brainstem [20], hippocampal glutamate neurons [21], dopamine (DA) neurons of the ventral tegmental area [22], NeuN positive neuronal cells hippocampus, postsynaptic somatodendritic areas, and NeuN negative (glia) cells (including microglia) [23]. The discovery of the functional neuronal CB2R raised new possibilities for the potential and safe targeting of the ECS for the treatment of neuropsychiatric and neurodegenerative disorders, including AD [24].

Although CB2R levels are low in the neuronal brain cells in healthy conditions, many studies have suggested that brain CB2Rs are inducible or upregulated in response to various insults, including multiple sclerosis [25], AD [26], chronic pain [27], ischemia-induced hypoxia, HIV-induced encephalitis, and drug addiction [18,24]. Indeed, brain CB2R levels significantly increase (up to 100 times) in the case of neuronal damage or inflammation. CB2R upregulation may be explained by the overexpression of the receptor or by the migration of immune cells that express CB2R in loco [28]. Following this discovery, significant research was carried out to assess the neuroprotective role of CB2R in the CNS. 

Concerning their downstream signaling pathways, both CB1R and CB2R preferentially couple to Gα_i/o,_ and their activation determines an intricate picture of cellular responses. One of these is the decrease in cyclic adenosine monophosphate (cAMP) accumulation triggered by the suppression of adenylyl cyclase (AC) activity, followed by regulation of cAMP-dependent enzymes, such as protein kinase A (PKA) [13]. Through the action of Gα_i/o_, CBRs can activate different members of the mitogen-activated protein kinase (MAPK) family, including extracellular kinase-1 and -2 (ERK1/2), p38 and p42/p44 MAPKs, and c-Jun N-terminal kinase (JNK), which mediate numerous cellular processes, such as the regulation of cell proliferation, mechanisms of differentiation, apoptosis, gene transcription, and cytokine release [15]. Through coupling to the G-protein Gβγ subunit, CB1R activation can modulate the phosphoinositide 3-kinase (PI3K)/protein kinase B (Akt) pathway to promote glycolysis and modulate cell proliferation [29], as well as directly stimulate the hydrolysis of phosphatidylinositol diphosphate (PIP2) by PLC-β (phospholipase C-β) with the subsequent release of inositol-1,4,5-triphosphate (IP3), Ca^2+^ mobilization, and activation of protein kinase C (PKC). Moreover, it has long been suggested that the CB1R regulates ionic fluxes by inhibition of N- and P/Q-type voltage-gated Ca^2+^ channels (VGCCs) [30] and stimulation of A-type and inward-rectifying K^+^ channels [31]. Compared to the CB1R, the CB2R uses fewer routes; indeed, this receptor has a smaller role in controlling ion channels but, like the CB1R, it increases ceramide levels, promoting the hydrolysis of sphingomyelin or the synthesis of ceramide de novo and, thus, modulating the gene transcription [32]. Furthermore, both CB1R and CB2R can interact with β-arrestin, which is the scaffold protein responsible for their desensitization and internalization processes [33].

## 2. Microglia: Functions and Phenotypes

Microglia, the resident immune cells of the brain, regulate the development and the homeostasis maintenance of the central nervous system (CNS). They are highly dynamic, and, depending on the microenvironment, they can adopt different activation states/phenotypes, becoming involved both in neuroinflammation and pro-resolutive inflammatory processes [34]. 

Often referred to as brain-resident macrophages, microglia dynamically survey the environment and perform important homeostatic functions, playing a crucial role in CNS tissue maintenance, injury response, and pathogen defense [35]. They interact with other immune cells (e.g., T cells), and release a vast array of pro- and anti-inflammatory cytokines and endogenous lipids, such as eCBs, arachidonic acid-derived autacoids, and pro-resolving mediators. Moreover, microglia cells participate in pivotal brain functions, such as synapse pruning and neuronal circuit remodeling, regulation of the synaptic neurotransmitter tone, elimination of apoptotic cells, misfolded proteins, and other cellular debris [36,37,38].

**Figure 1:**
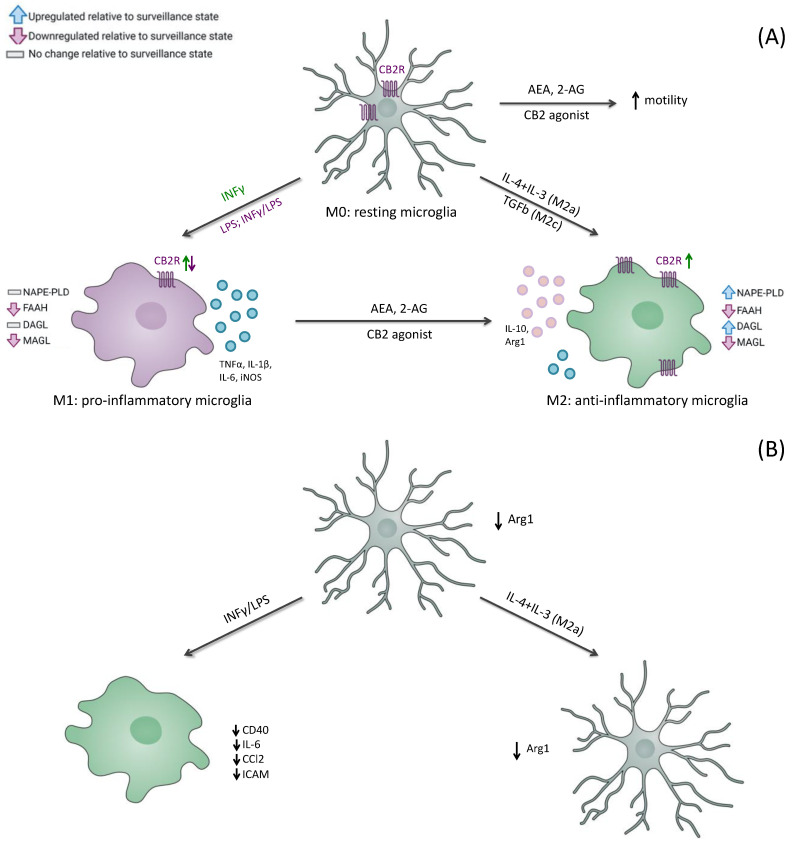
CB2R and microglial polarization. (**A**) CB2R activation in resting microglia (M0) results in increased microglial motility. Activation of microglia (M1, pro-inflammatory microglia) by IFN-γ increases CB2R expression, whereas activation by a combination of IFN-γ/LPS or LPS decreases CB2R expression and increases the secretion of pro-inflammatory cytokines (e.g., TNFα, IL-1β, iNOS). Stimulation of CB2R with agonists causes a switch from the M1 to M2 phenotype (anti-inflammatory microglia). M2 microglia upregulate CB2R, decrease the secretion of pro-inflammatory cytokines, and increase the expression of anti-inflammatory cytokines (e.g., IL-1β, Arg1). (**B**) Deletion of microglial CB2R leads to a suppression of the pro-inflammatory phenotype. Stimulation of microglia with IFNγ/LPS decreases the secretion of pro-inflammatory cytokines and the expression of inflammatory markers. Similarly, alternative activation of microglia by IL-4 + IL-13 does not take place, as Arg1 remains decreased. Small arrows represent the direction of the effects: increase (↑) or decrease (↓). The green arrow refers to an increase in CB2R, and the purple arrow refers to a decrease in CB2R.

Under physiological conditions, microglial cells exhibit a “resting” or “M0” phenotype, maintaining a highly ramified morphology with highly branched processes, which allows them to constantly monitor and protect neuronal functions [39,40] (Figure 1A).

While the soma remains relatively stationary, microglia cells continually extend and retract their numerous processes to survey the CNS environment. Constant interaction with neurons and other glial cells, either through direct contact or through secreted mediators, allows microglia to detect changes, such as brain injury and infection, ischemia, inflammatory mediators release, and changes in ion gradients, that may put physiological homeostasis at risk [41]. Furthermore, their immune pattern recognition receptors (PRRs), represented by Toll-like receptors (TLRs), Nod-like receptors (NLRs), C-type lectin receptors (CLRs), and RIG-like receptors (RLRs), allow them to recognize any molecular signals that indicate cell damage or stress, including ATP, nucleic acids, necrotic cells, cell debris, and misfolded proteins, such as β-amyloid [42,43,44]. When detected, these ligands are internalized, and homeostatic microglia may shift to amoeboid phenotypes, characterized by an enlarged cell body and shorter processes, which mediate neuroinflammatory reactions through the secretion of cytokines and chemokines. Morphological changes in activated microglia also accompany functional responses, such as phagocytosis, migration, and antigen presentation [45]. 

In detail, depending on the nature of the stimulus, microglia can adopt two different phenotypes, commonly classified as pro-inflammatory, or M1-like, and pro-homeostatic, or M2-like, microglia (Figure 1A) [46].

Tissue injury and neurodegeneration can determine the assumption of a pro-inflammatory phagocytic phenotype, defined as thr “M1 state”, characterized by an amoeboid form, with retracted processes and an enlarged cell body. M1-like microglia cells play a fundamental role in inducing innate immune responses to fight foreign pathogens and trigger the adaptive immune response. Pro-inflammatory microglia activate transcription factors, which trigger the upregulation of pro-inflammatory cell surface markers. Additionally, they induce the release of pro-inflammatory mediators, including cytokines (e.g., tumor necrosis factor α (TNF-α) and various interleukins (IL), such as IL-1β, IL-6, IL-12, IL-17, IL-18, IL-23, chemokines (e.g., Chemokine (C-C motif) ligand 12 (CCL12) and ligand 10 (CXCL10)), and reactive oxygen and nitrogen species (ROS and NOS, respectively) [42,43,44,45,46,47]. However, the chronic pathological activation of microglia cells contributes to exacerbating neuroinflammation, oxidative stress, and neurotoxicity [48].

Microglia are capable of dynamically shifting between M1-like and M2-like phenotypes. As a consequence of brain homeostasis disruption, due to brain injury or chronic stress, the CNS promotes tissue repair by releasing anti-inflammatory cytokines (IL-4, IL-10, IL-3), growth factors (TGF-β), and hormones, and promotes surrounding microglia to transform into an M2-like protective phenotype. Thus, the initial activation of the M1 state is followed by the alternative M2 state, which mostly mediates anti-inflammatory and neuroprotective functions through the secretion of anti-inflammatory and neurotrophic factors, playing a pivotal role in immune resolution and tissue repair [47,48,49].

## 3. Alzheimer’s Disease and Microglia

Alzheimer’s disease (AD), a multistage neurodegenerative disorder affecting mainly the elderly population, accounts for almost two-thirds of the worldwide cases of dementia and cognitive decline. It is characterized by massive neuronal death, brain atrophy, loss of neurons, and synaptic dysfunction, which all lead to a subsequent progressive cognitive decline [50]. 

AD progresses through distinct stages, each reflecting a different level of cognitive and functional impairment. The clinical phases of AD can be broadly classified as follows: preclinical phase, in which subacute brain abnormalities may be present but without obvious symptoms; mild cognitive impairment, marked by cognitive and memory issues; mild AD, characterized by difficulties to assess everyday duties due to growing memory and cognitive deficiencies; moderate AD, with a worsening of cognitive deterioration, changes in personality and behavior, and trouble in basic self-care; severe AD, the terminal phase of the disease, characterized by an overt dementia that results in complete cognitive decline, physical incapacity, and death from immobility [50]. Cannabinoid-based therapy may be beneficial in both the early and advanced stages of the pathology. In particular, CBD-rich preparations could have greater potential for prophylactic use in preclinical, prodromal (e.g., mild cognitive impairment), or mild AD cases. Conversely, Δ^9^-THC-rich formulations may be more relevant for managing advanced stages of the disease [51].

Historically, the cause of the neurodegeneration has been linked to amyloid plaques, which are extracellular deposits of amyloid-β peptide (Aβ) aggregates, and intracellular neurofibrillary tangles (NFTs), generated by the accumulation of abnormal filaments of tau protein in brain regions that serve memory and cognition [52].

A crucial aspect of Alzheimer’s pathology is cholinergic deficiency, which means that the neurotransmitter acetylcholine is not functioning properly. Cholinergic neurotransmission is essential for functions, such as learning, memory, sleep, and stress regulation. Dysfunctions in this system are associated with neuroinflammation and neurodegeneration, which are characteristic features of neurodegenerative diseases, like AD [50]. A large body of evidence indicates that acetylcholinesterases (AChE) are able to promote Aβ aggregation with the formation of highly toxic complexes, that displayed a neurotoxic effect higher than that produced by Aβ peptide alone. Currently, inhibitors of AChE represent the most commonly used treatment for improving the cognitive symptoms of AD. [53,54].

Notably, selected CB modulators have been tested for their ability to inhibit AChE [53,55]. For instance, Δ^9^-THC has been shown to competitively inhibit AChE, increasing acetylcholine (Ach) levels and preventing AChE-induced Aβ aggregation [55].

In recent years, neuroinflammation emerged as a central cause of neuronal loss in AD, with microglial cells considered to have the main role in this process [56].

Current evidence suggests that neuroinflammation has a vital role in the pathogenesis and progression of AD, underling the fundamental involvement of overactivated or dysfunctional microglia in AD pathophysiology. Indeed, microglia cells were found colocalized with amyloid plaques in the brains of people affected by AD [42], and microglial activation has been reported in animal models even before the formation of amyloid plaques [57], suggesting a strong and early interaction between Aβ and microglia. 

Furthermore, in support of the role of early neuroinflammation in AD, variants of highly expressed microglial genes (e.g., triggering receptor expressed on myeloid cells 2 (TREM2), CD33, membrane spanning 4-domains A6A (MS4A6), and ATP binding cassette subfamily A member 7 (ABCA7)), which mediate important functions of microglia, have been identified as risk factors for the development of AD. In particular, the triggering receptor expressed on myeloid cells 2 (TREM2), whose signaling promotes microglial proliferation, phagocytosis, and cytokine secretion, is highly expressed in plaque-associated microglia in patients with AD [58,59].

Interestingly, recent evidence revealed that the presence of Aβ is an enhancer of microglia activation, because microglial cells recognize amyloid and tau aggregates as pro-inflammatory stimuli, leading to morphological changes, and production of pro-inflammatory mediators [42]. 

In addition, a new microglial phenotype, known as “dark microglia”, has been described as abundant during chronic stress, aging, and AD. Dark microglia exhibit several signs of oxidative stress, including condensed cytoplasm and nucleoplasm, which result in their dark appearance in electron microscopy [60]. Dark microglia appear to be extremely active and express high levels of the myeloid cell marker CD11b, which forms the phagocytic receptor CR3, TREM2, and the recently discovered marker of homeostatic microglia 4D4 [60].

## 4. Endocannabinoid Signaling and Microglia Activation 

The ability of endocannabinoid signaling to control microglial activity makes the ECS a powerful orchestrator in the prevention and treatment of CNS dysfunction. This evidence led to the assumption that microglia possess a complete ECS, whose components greatly change between the different pro-inflammatory and anti-inflammatory microglia phenotypes (Figure 1B).

Resting microglial cells (M0 phenotype) produce both 2-AG and AEA at a low rate and become a major cellular source of eCBs under neuroinflammatory conditions. In particular, Walter and colleagues (2003) observed that microglia can release 20 times more eCBs than neurons and astrocytes [61]. These findings made an important contribution to the understanding of the role of cannabinoid signaling in the management of microglia function. In detail, pathological overstimulation of neurons induces 2-AG synthesis and attracts adjacent microglial cells, eventually leading to neuron death. When these microglial cells reach the site of injury, ATP produced by the dying neurons interacts with the nearest microglial cells, increasing local 2-AG (without affecting the amount of other eCBs) and recruiting more microglial cells [62]. The increased release of 2-AG by both neurons and microglia and consequent further microglia recruitment participate in the establishment of a loop propagating the pathological dysfunction.

In addition, eCB synthesis strongly depends on the different microglia phenotypes. Indeed, Carrier and co-workers demonstrated that, apart from ATP, the ionomycin is another ‘stimulus’ that leads to a more substantial and selective increase in 2-AG than AEA [63].

Furthermore, further evidence supports the idea that eCB synthesis is closely linked to the state of microglial activation by demonstrating how 2-AG expression increases in the brain of a mouse model of experimental autoimmune encephalomyelitis (EAE) [64]. This study builds on important previous evidence produced by the same research group, which unveiled that ATP-induced 2-AG production, previously described by Walter and colleagues, relates to the activation of the purinergic P2X7 receptor (P2X7R) [65]. P2X7R is highly permeable to calcium ion (Ca^2+^) and, in response to elevated intracellular levels of this ion, an increase in DAGL activity (deputy for 2-AG synthesis) and a decrease in MAGL activity (deputy for 2-AG degradation) were induced [66]. In light of this finding, in a subsequent work, Witting and co-workers observed that P2X7R knockout mice showed reduced 2-AG production compared to wild-type mice, thus, reinforcing the hypothesis that 2-AG synthesis is effectively mediated by P2X7R [67].

Beyond the changes in the concentration of eCBs, cultured microglial cells have also been observed to vary in the expression of the full assortment of synthetic and degrading enzymes for eCBs. In particular, the pro-inflammatory microglial state (M1 phenotype) is characterized by an unaltered expression of the biosynthetic enzymes, such as DAGL and NAPE-PLD, and a reduced expression of the catabolic enzymes, such as MAGL and FAAH, as compared to the surveillance microglial state (M0 phenotype) (Figure 1). On the other hand, it has been observed that the microglia’s switch to the relatively anti-inflammatory and protective M2 phenotype leads to an upregulation in biosynthetic enzymes, and a concomitant downregulation in metabolic enzymes, resulting in increased production of eCBs (Figure 1). Therefore, the differences in the expression of the enzymatic machinery of eCBs depending on the microglial activation states, make these ligands potential therapeutic tools in neuroinflammation [66].

Different microglia phenotypes also result in a significant fluctuation in the density of CBRs. It is generally accepted that the abundance of CB1R and CB2R is relatively low in resting microglia since the mRNA encoding for these receptors is only detectable in trace amounts in healthy brain tissue [67,68].

Although early reports indicated that the CB1R was neuron-specific, it was later shown to be constitutively present in microglia [69], and was subsequently identified as the endocannabinoids target receptor in resting microglia. Notably, the abundance of this receptor subtype does not appear to show any significant change in the different microglia states, since its mRNA transcripts were found to be only slightly overexpressed (2-fold) in LPS-activated microglia [69].

Conversely, the expression of the CB2R varies considerably between the different phenotypes of activated microglia following specific neuroinflammatory responses, and it is strongly correlated with the type of stimuli [70]. For instance, it has been shown that pro-inflammatory stimuli, such as granulocyte-macrophage colony-stimulating factor (GM-CSF) and interferon-γ (IFN-γ), cause an increase in CB2R mRNA in microglia [71]. On the other hand, activation of cultured microglia cells with LPS or LPS/IFNγ was observed to reduce the expression of CB2R [71,72].

Pharmacological or genetic methods were employed to investigate how CB2R affects microglial polarization from an inflammatory M1 activation state to an anti-inflammatory M2 activation state [73].

In vitro pharmacological studies showed that CB2R activation increases motility in the resting microglia (M0 state). In the presence of an inflammatory stimulus, pharmacological activation of CB2R by endo- or exogenous agonists drives the switch of microglia from M1 to M2 state, causing a decreased release of pro-inflammatory cytokines, such as TNFα, IL-6, IL-1β and iNOS [74,75].

CB2R stimulation results not only in reducing the secretion of pro-inflammatory mediators but also in increasing the expression of anti-inflammatory cytokines [56,76].

The correlation between CB2R and microglia state was further described by Mecha and colleagues (2015) [77], who analyzed the ability of microglia to acquire diverse states of activation in response to activation/inhibition of CB2R both in in vitro and in vivo models. These authors reported that, after 6h induction of the pro-inflammatory M1 phenotype, the expression of ECS machinery (including CB2R, NAPE-PLD, FAAH, and MAGL transcripts) was significantly reduced. As opposed to the M1 phenotype, when microglial cells were stimulated with anti-inflammatory cytokines, such as IL-4 and IL-13, significant changes in the expression of the main components of the ECS were observed. In detail, CB2R expression was upregulated, as well as the expression of DAGLα isoform, which produces 2-AG, whereas a significant downregulation of the enzymes that degrade eCBs, namely FAAH and MAG, was observed [77]. Moreover, the effects of the genetic ablation of the microglial CB2R in vivo model, (CB2R-deficient mice, named CB2^−/−^) resulted in a decrease in the inflammatory phenotypes. Indeed, microglia from CB2^−/−^ mice revealed a lower expression of arginase 1 (Arg1) after stimulation with IL-4/IL-13, indicating that they were unable to polarize to an M2 phenotype [77].

In summary, the activation of CB2R in microglial cells results in an increased expression of M2 anti-inflammatory marker, associated with a decreased M1 pro-inflammatory marker expression, whereas the inhibition of CB2R produces opposite effects.

## 5. Cannabinoid Receptors-Microglia Communication: Therapeutic Implications for Alzheimer’s Disease

In recent years, the ECS has emerged as a promising strategy for treating AD in its early stages. During the preclinical phase of the neurodegenerative process, significant pathological events take place, including protein misfolding, neuroinflammation, excitotoxicity, mitochondrial dysfunction, and oxidative stress. Treatments focusing on a single target in light of these mechanisms have shown limited efficacy. However, cannabinoids exhibit a pleiotropic activity, simultaneously addressing key stages in AD, such as aberrant processing of Aβ and tau, neuroinflammation, excitotoxicity, mitochondrial dysfunction, and oxidative stress. Therefore, they are promising drugs for the treatment of neurodegenerative disorders [53]. 

The first cannabinoid recognized for its ability to interfere with neurodegenerative processes was Δ^9^-THC, the most abundant compound in marijuana extract with a similar affinity for both CBR1 and CB2R. This cannabinoid displayed significant therapeutic potential for the treatment of neurodegenerative diseases, such as AD. Evidence reported its ability to interfere with Aβ aggregation in vitro, influence Aβ fibrils formation and aggregation, stimulate the removal of intracellular Aβ, and block the inflammatory response. Additionally, THC has been shown to inhibit AChE activity more effectively than approved drugs for AD treatment, such as donepezil and tacrine. However, the primary limitations of THC use in clinical practice stem from its psychoactive effects, which result from the activation of CB1R, including a reduction in cognitive functions, learning, memory, attention, and executive function [78]. To date, the only FDA-approved chemical modification of Δ^9^-THC is nabilone, marketed as Cesamet, used for the treatment of nausea and vomiting associated with cancer chemotherapy [79].

In recent decades, several synthetic cannabinoid compounds have undergone testing as therapeutic tools in various preclinical models, including in vitro and in vivo AD models.

### 5.1. CB1R-Mediated Effects on Microglia Activation In Vitro and In Vivo Models

Regarding the involvement of CB1R in the regulation of microglial function and neuroinflammation, extensive evidence has been produced, but the precise mechanism remains to be understood [80]. In experimental models of AD, CB1R has been detected as a suitable target for CB1R agonists inducing repair mechanisms and protection against tau phosphorylation and Aβ action [53]. Unfortunately, the therapeutic application of CB1R agonists has been limited because of the psychotropic effects produced [81]. 

On the contrary, other studies reported the use of CB1R antagonists as effective anti-inflammatory drugs, even though some evidence highlights that CB1R activation can shift microglial phenotype toward an anti-inflammatory/prophagocytic profile in vitro. Indeed, AM251, a CB1R selective antagonist, was able to reverse the increase in Arg1 mRNA and protein in microglia cells treated with IL-4 and IL-13, demonstrating that the activity of CB1R is also important to enable the transition from an unreactive to an M2 phenotype [77]. In the study by Lou et al. (2018) [82], SR141716A, a CB1R antagonist, favored the release of inflammatory factors (TNF-α, IL-1β, IL-6) in BV-2 microglia while inhibiting the production of IL-10 and chemokines (MCP-1, CX3CL1). Furthermore, when splenic CD4+ T cells were co-cultured with SR141716A-administered BV-2 microglia, a decrease in IL-4 and IL-10 and production of IL-17 and IFN-γ were detected. De Meij et al. (2021) [80] performed different experiments in mice deficient in microglial CB1R (CX3CR1-CB1R-KO), demonstrating that in CX3CR1-CB1R-KO mice the exposure to an immune challenge induced a decrease in the production of central proinflammatory cytokines as compared to wild-type mice (WT), thus, suggesting a CB1R proinflammatory role. Overall, these findings are in accordance with many previous reports showing that inhibition of CB1R activity, either in vitro or in vivo, protects cells from inflammation, dampening the brain production of pro-inflammatory cytokines. In Table 1, the CB1R modulator-mediated effects on microglia activation in AD in vitro models are shown. Notably, even though some contradictions can be deduced from this large body of studies, in a recent report, Navarro and co-workers underlined that the effects of cannabinoids on microglia may be qualitatively different depending on the stage of cell activation, and that, consequently, it is relevant to consider these different effects to find the most appropriate therapeutic window to allow their action [68]. 

**Table 1:** CB1R-mediated effects on microglia activation in vitro and in vivo models.

Model	CB1R Modulator	Outcome	Reference
Primary microglia cells isolated from P0-P2 Wistar rats	AM251 (antagonist)	↓ Arg1	[77]
Murine BV2 microglial cells	SR141716A (antagonist)	↑ TNF-α, IL-1β, IL-6 ↓ IL-10, MCP-1, CX3CL1	[82]
Co-culture of murine BV2 microglial cells and CD4+ T cells	SR141716A (antagonist)	↑ IL-17, IFNγ ↓ IL-10, IL-4	[82]
APP/PS1 mice Primary cultures of cortical neurons isolated from OF1 mice	Arachidonyl-2-chloroethylamide (ACEA) (agonist)	↓ Cognitive impairment ↓ Cytotoxic effect of Aβ_42_ olygomers	[53]

### 5.2. CB2R-Mediated Effects on Microglia Activation In Vitro and In Vivo Models

**Figure 2:**
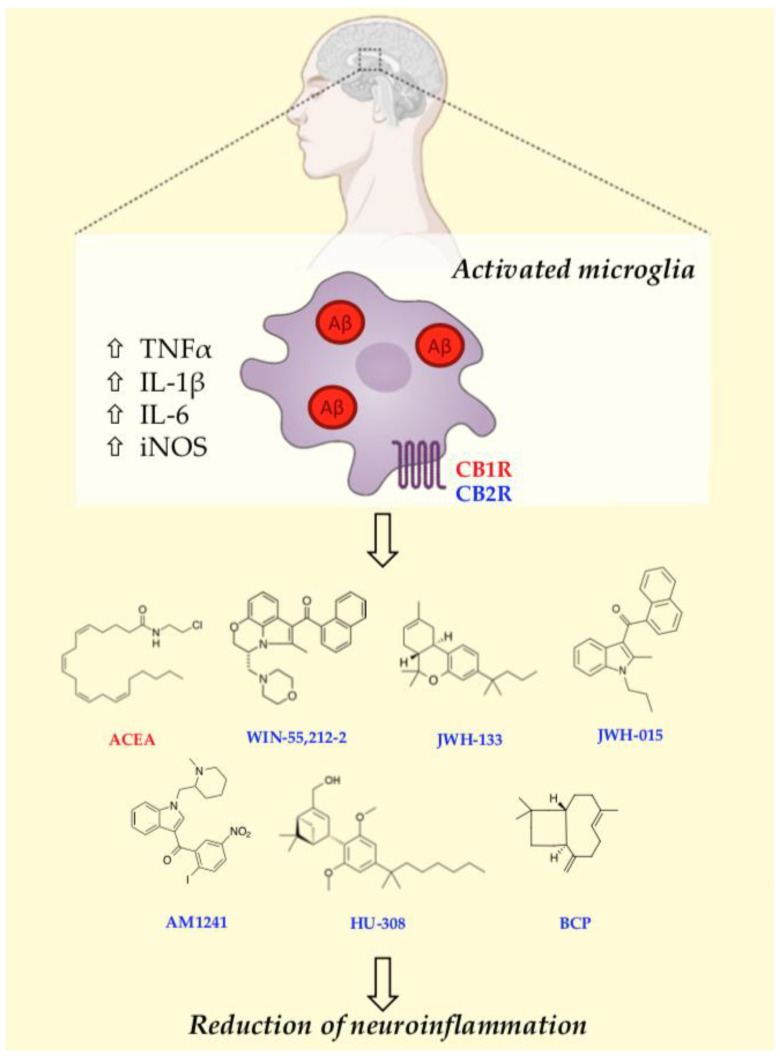
Schematic representation of anti-inflammatory and neuroprotective actions of CB modulators. The pharmacological activation of central CB receptors with CB1R (red), and CB2R (blue) agonists is a promising therapeutic approach for the treatment of AD, since it promotes anti-inflammatory and neuroprotective effects.

Given that CB1R is predominantly associated with the psychotropic effects of cannabinoids, CB2R emerges as an attractive pharmacological target. Numerous studies, primarily focusing on CB2R, have underscored its direct involvement in the anti-AD beneficial effects of cannabinoid-based therapies. Specifically, the role of microglial CB2R has been extensively examined in both in vitro and in vivo studies (Table 2), highlighting the fundamental function of activated CB2R in protecting microglia (Figure 2). Indeed, several preclinical studies conducted in animal models, despite occasionally conflicting results, consistently suggest their positive impact on memory and learning processes, as well as on other neurobiological mechanisms underlying AD.

#### 5.2.1. WIN55,212-2

**Figure 3:**
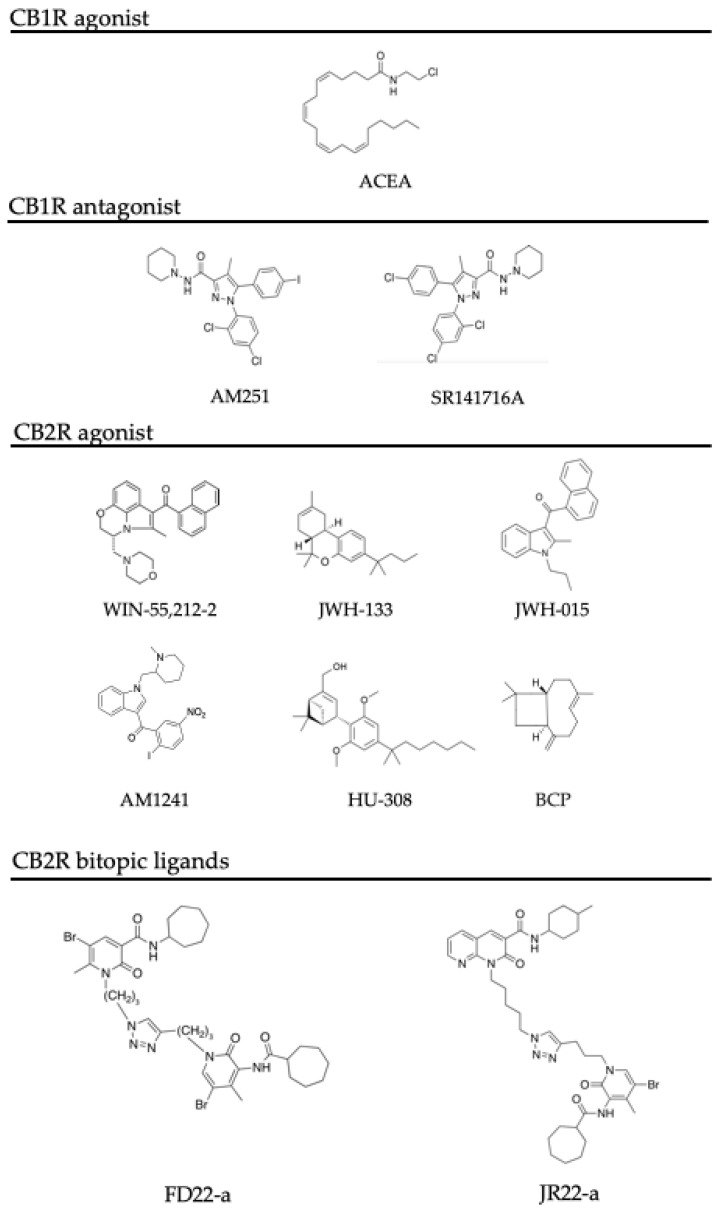
Chemical structures of CBR ligands involved in the modulation of microglia activation.

One of the first studies on WIN55,212-2 (Figure 3), a non-selective agonist of CB1R and CB2R, was conducted in 2003 by Facchinetti and co-workers, in which they demonstrated that WIN55,212-2 activity on CB2Rs inhibited the release of TNF-α in LPS-activated rat microglia cultures [83]. Later in 2009, it was demonstrated that 3 weeks of administration of WIN55,212-2 partially restored neurogenesis in the hippocampus of aged rats. However, in this study, the authors did not investigate if the effects were due to the stimulation of one or both CBRs [84]. In 2012, WIN55,212-2 was studied for its relevance in AD, and it was demonstrated to promote primary microglia migration [85]. Interestingly, this activity was reversed by both CB1R and CB2R antagonists, showing that its effect was due to its action on both receptors [86]. More recently, in 2016, WIN55,212-2 was studied in vivo in a rat model of essential tremor, revealing a suppression of tremor, cognitive impairment, and anxiety [87]. 

#### 5.2.2. JWH133 and JWH015

JWH133 (Figure 3) is a selective CB2R agonist. In microglial cells, it was shown to upregulate the microglia M2-phenotype markers TGF-β, IL4, IL-10, CD206, and Ym1. The selectivity of CB2R was assessed by using a selective CB2R antagonist, which was demonstrated to completely reverse the activity. Ramirez et al. demonstrated that 100 nM of JWH133 was able to prevent Aβ-induced microglia activation in microglial cells treated for 4 h with 0.5 μM fibrillar Aβ [88]. In an in vivo model of Alzheimer’s disease (i.e., an AβPP/PS1 transgenic mouse model), treatment with JWH133 (200 nM) has been shown to lead to cognitive improvement, the results of which were associated with decreased microglial reactivity and a reduced expression of pro-inflammatory cytokines IL-1β, IL-6, TNFα, and IFNγ [89]. More recently Chung and co-workers demonstrated that JWH133 was able to suppress the production of proinflammatory factors in rats, such as IL-6, TNFα, and iNOS [90].

JWH015 (Figure 3) is a selective CB2R agonist. In the already mentioned study conducted by Ramirez and co-workers, JWH015 was demonstrated to be able to counteract the increase in TNF-α in microglial cultures treated with β-amyloid fibrils [88]. Moreover, JWH-015 suppressed IFN-γ-induced CD40 expression and markedly inhibited IFN-γ-induced phosphorylation of JAK/STAT1. Additionally, the same ligand was shown to suppress microglial TNF-α and nitric oxide production induced either by IFN-γ or Aβ peptide challenge in the presence of CD40 ligation. In the same study, it was assessed that CB2R activation by JWH-015 markedly attenuated CD40-mediated inhibition of microglial phagocytosis of Aβ1–42 peptide [74]. In the study conducted by Li and colleagues, treatment of transgenic APP/PS1 mice with JWH-015 displayed a significant impact on cerebral region-specific regulation of microglia phenotype transition from the M1 to M2 phenotype and dendritic complexity, concomitant with a corresponding region-specific modulation in cognitive abilities. Indeed, the results of the study showed that after treatment of APP/PS1 mice with JWH-015, CB2R activation normalized cortex-dependent memory deficit, but the treatment was ineffective for hippocampus-dependent spatial cognitive dysfunction. Notably, in the cortex but not in the hippocampus, JWH-015 treatment significantly promoted M1 to M2 microglial phenotype conversion, enhancing mRNA expression of M2 microglia biomarkers Ym1/2 and suppressing mRNA expression of M1 microglia biomarkers (IL-6, iNOS, and TNF-α), and improved the dendritic complexity [91]. Taken together, these behavioral and molecular results suggest a potential role of CB2R as a pharmacologic target for AD.

#### 5.2.3. AM1241

AM1241 (Figure 3) is a selective CB2R agonist. A study by Ma and co-workers on rat primary microglia cells N9 reported that 10 µM AM1241 was able to reduce LPS (10 ng/mL)/IFN-γ (10 U/mL)-induced microglial activation by switching the phenotype of microglia from the M1 to M2 state [75], indicating its ability to counteract the AD-related inflammatory response.

These neuroprotective effects were confirmed by a recent in vivo study, where AM1241 demonstrated a significant restoration of learning and memory in APP/PS1 mice. This was achieved through the suppression of Aβ plaque deposition, facilitation of Aβ phagocytosis, and promotion of neurogenesis [92].

#### 5.2.4. HU-308

HU-308 (Figure 3) is a selective CB2R agonist. Several studies on in vivo models of neurodegeneration showed a decrease in microglia proliferation and cytokine expression, and an improved neuroprotection after the treatment with HU308 [93].

#### 5.2.5. β-Caryophyllene

β-Carophyllene (BCP, Figure 3) is a dietary selective CB2R agonist, with abundant presence across cannabis and non-cannabis plants, including spices and other edible plants. As recently reviewed by Ullaha et al. [94], a large body of literature suggests that BCP possesses a neuroprotective capability through decreasing oxidative stress and stabilizing mitochondria, and it could be a potential lead molecule in the discovery of drugs for neurodegenerative disorders. In a recent report, Askari et al. [95] investigated the protective effects of a broad range concentration of BCP against LPS-induced primary microglia cells inflammation and M1/M2 imbalance, highlighting that the protective effect of BCP was provided by the M2 healing phenotype of microglia, releasing the anti-inflammatory (IL-10, Arg-1, and urea) and anti-oxidant (GSH) parameters and reducing the inflammatory (IL-1β, TNF-α, PGE_2_, iNOS and NO) and oxidative (ROS) biomarkers. 

More recently, the same group has further explored the CB2R-mediated neuroprotective activity of BCP by using experimental autoimmune encephalomyelitis (EAE) mice as a chronic MS model. According to their findings, low doses of BCP resulted in offering a CB2R-dependent protective impact in the EAE mice treatment, by simultaneously targeting both adaptive (lymphocytes) and innate (microglia) immune systems, ultimately leading to the resolution of inflammatory processes. In particular, the authors confirmed the previously reported ability of BCP to reduce the levels of pro-inflammatory factors (TNF-α, IL-1β, PGE2, NO, ROS, INOS), to increase the levels of anti-inflammatory Arg-1, IL-10, and urea, and to promote the polarization of microglia cells to the M2 anti-inflammatory phenotype [96].

These findings further corroborate the therapeutic potential of BCP in the treatment of multiple inflammatory neurological diseases.

#### 5.2.6. RO6866945

RO6866945 is a novel selective and brain-penetrant CB2R agonist synthesized by Roche. Esteban et al. treated 5xFAD/^CB2EGFP/f/f^ and 5xFAD/CB2^−/−^ male mice with this agonist, and since microglia are the main source of cannabinoid CB2R in the brain of the mice, putative changes triggered in these cells by the activation of the CB2R and by its genetic deletion were analyzed. A decrease in Iba1+ microglia abundance as well as an impairment in its phagocytic activity compared to knock-out mice was detected, but the major changes were in in cAMP, CREB, and p38MAPK signaling cascade. Notably, it was highlighted how p38MAPK is under CB2R control since its activity was significantly reduced in knockout mice [97]. These results, especially microglial phagocytosis and signaling cascade (p38MAPK) profiles, are in agreement with those previously published by Reusch et al. [98].

**Table 2:** CB2R-mediated effects on microglia activation in vitro and/or in vivo models.

Model	CB2R Modulator	Outcome	Reference
Rat (Aβ_25-35in_j)	WIN55,212-2	↓ Microglia activation	[88]
Tg2576 mice	WIN 55,212-2 and JWH-133	↓ Microglial cell density was decreased by continuous JWH-133 oral treatment. ↓ COX-2 and TNF-α ↓ Aβ cortical levels	[85]
APP/PS1 mice	JWH133	↓ Microglial activity ↓ IL-1β, IL-6, TNF-α, and IFNγ secretion	[89]
	JWH015	↓ Expression of M1 microglia biomarkers (IL-6, iNOS and TNF-α) ↑ Expression of M2 microglia biomarkers Ym1/2	[91]
Murine N9 microglial cells APP/PS1 mice	AM1241	↑ Arg1/IL-10/BDNF/GDNF↓ iNOS/IL-1β/IL-6/TNFα ↓ Amyloid plaque deposition ↑ Aβ phagocytosis	[75] [92]
R6/2 mice	HU-308	↓ Proliferation of microglia and cytokine expression ↑ Neuroprotection	[93]
Primary microglia cells isolated from C57BL/6 mice	β-Caryophyllene	↓ Expression of M1 microglia biomarkers (IL-1β, iNOS, TNF-α, NO, ROS) ↑ Expression of M2 microglia biomarkers (IL-10, Arg-1, and urea, GSH)	[95,97]
Microglial cells isolated from 5xFAD/CB2EGFP/f/fmice	RO6866945	↓ Iba1+, phagocytosis activity ↑ cAMP, CREB and p38MAPK	[97]

### 5.3. Potential Influence of Microglial CB1R-CB2R Heteromer-Mediated Regulation in Alzheimer’s Disease

The heteromerization of GPCRs is a well-accepted phenomenon. In 2012, Callen et al. demonstrated for the first time that CB1R and CB2R also form heteromers in neuronal cells and the brain. A negative cross-talk between the two receptors and a bidirectional cross-antagonism phenomenon were uncovered as specific characteristics of CB1-CB2 receptor heteromers [99]. In 2018, Navarro and co-workers investigated the expression and signaling properties of CBRs both in resting and in LPS/IFN-γ-activated microglia and found an increased expression of CB_1_-CB_2_ receptor heteromers (CB1R-CB1RHets) in activated microglia, which also resulted in them being highly responsive to cannabinoids. Similar results were obtained in cultures treated with β-amyloid (Aβ_1-42_), and in primary cultures of microglia from APP_Sw,Ind_ mice, a transgenic AD model [68]. The notable increase in the expression of CB1R-CB2RHets in activated microglia makes these complexes a target when designing therapeutic approaches toward alterations involving the endocannabinoid system, such as neurodegenerative/neuropsychiatric disorders.

### 5.4. Current Hot Topics in CBR-Oriented Drug Discovery: Allosteric and Bitopic Modulation

Until now, cannabis and cannabis-derived compounds have not been approved by the US Food and Drug Administration (FDA) for the treatment of AD. Only a small number of clinical trials evaluating the use of CBD or THC (dronabinol and nabilone) have been completed or are still underway [78]. One of the main limitations hampering the success rate of CBR-targeted agents in AD therapy is the non-specificity of their mode of action. The concept of biased agonism (e.g., functional selectivity) can be adopted to closely monitor cannabinoid pharmacology in microglial cells and, subsequently, to create novel and safer therapeutic platforms to treat and manage AD. Biased ligands promote ligand-dependent selectivity for certain signal transduction pathways over a native ligand of the same receptor, providing a potentially valuable framework for developing novel therapeutics with minimized side effects [100]. 

For instance, recent evidence suggests MAPK pathways as potential targets to explain how CB2R agonism may inhibit pro-inflammatory microglia activation [73]. In this context, a recent report found that CB2R-knockout microglia exhibit impaired MAPK signaling paths, consistent with the role of CB2Rs in these pathways [98]. Therefore, although the specific mechanisms and signaling pathways by which cannabinoids and cannabinoid-like drugs manipulate microglial activity have not been thoroughly examined, it is reasonable to assume that potential CB2R ligands that have a bias toward the activation of these intracellular signaling pathways may promote an important control of microglial activity.

A convenient way to offer superior functional selectivity, as well as greater receptor subtype selectivity, compared to conventional CBR modulators, may be allosteric ligands. Allosteric modulators (AMs) have no intrinsic activity but can remotely modulate receptor activity through endogenous or exogenous ligands that induce activity at the binding site [101]. They can increase, decrease, or leave unaffected the response of the orthosteric agonist, providing positive, negative, or neutral modulation. Theoretically, they could represent a valuable alternative to orthosteric modulators because they could provide fine-tuning of microglial CBR signaling and, because of the development of cannabinoid-based drugs for the treatment of AD, would be associated with fewer unwanted effects and an effective pharmacological profile [102].

The prospects of interrogating allosteric sites in drug discovery were investigated for the CB2R only a few years ago when, in 2019, Manera and her team published the first synthetic positive AM (PAM) of the CB2R, named EC21a [103]. Its PAM profile has not only been ascertained through functional studies but also emerged in studies conducted on LPS-activated mouse BV-2 microglial cells in combination with an orthosteric CB1R/CB2R agonist, whose ability to modulate the release of pro- and anti-inflammatory interleukins (IL-6 and IL-10, respectively) was enhanced by EC21a [101]. Another trend in drug discovery that may offer a potential strategy for designing CBR-directed therapies for AD is bitopic modulation. Bitopic ligands simultaneously target orthosteric and allosteric sites of the same receptor and, consequently, combine the activation properties and the high affinity typical of orthosteric ligands with the higher selectivity profile of allosteric ligands. Additionally, they are independent of the presence of a correct endogenous agonist tone, which can occasionally fail, as in the case of neurological disorders. This makes them different from conventional allosteric modulators [104]. This approach has been recently applied to the cannabinoid research field, resulting in the development of the first bitopic CB2R ligand, FD-22a [105]. Actually, research in this field continued until the discovery of the second bitopic CB2R ligand, JR-22a, which provided new insights into the dual orthosteric/allosteric stimulation of CB2Rs [106,107].

These findings have also encouraged studies concerning the impact of CB2R bitopic modulation on microglial activity. In particular, our in vitro results indicated that both FD-22a and JR-22a contrast the inflammatory process in microglial cells [105,106]. Additionally, to obtain a deeper understanding of FD22a neuroprotective properties, our research group is currently investigating whether FD22a could promote the induction of autophagy in neuronal cells whose dysfunction has been directly linked to a growing number of adult-onset neurodegenerative disorders.

## 6. Conclusions 

Brain homeostasis depends crucially on microglia. In fulfilling their role, these cells assume a somewhat ‘chameleon-like’ attitude, acquiring heterogeneous phenotypic states depending on the brain region and the pathological condition, as well as its state of progression. Evidence convincingly demonstrates the existence of endocannabinoid signaling in microglial cells, which not only express CBRs, but are also able to synthesize and metabolize eCBs. As a result, there is close communication between the components of the ECS and microglia that can be manipulated for therapeutic benefits to counteract various neurodegenerative disorders. Among these, the studies outlined in this review focus on the role of CBRs in regulating microglial phenotypes and activity under AD-driven pathological conditions. The ECS may be directly responsible for the development of a microglial anti-AD phenotype that includes improved phagocytosis, chemotaxis, and the production of anti-inflammatory and pro-resolving lipid mediators. 

While there is still some controversy regarding the effect of microglial CB1R activation in AD-related circumstances, the direct involvement of microglia in anti-AD effects is particularly relevant to the CB2R. Most of our knowledge on CB2R-mediated microglial signaling in AD is still based on in vitro and in vivo research. Although an increasing number of preclinical studies have shed light on the beneficial and pleiotropic effects exerted by CB2R stimulation in AD rodent models, it is still unclear whether all these effects can be attributed to a tangible impact on microglia activity.

Thus, there is still much to learn about the molecular mechanisms that underlie the role of CBRs in the modulation of AD-mediated microglia activation. This knowledge will be fundamental to the CBR-based AD drug development process, which may even be extended to include modern medicinal chemistry paradigms, such as allosteric and/or bitopic modulation.

## Data Availability

No new data were created in this study.
